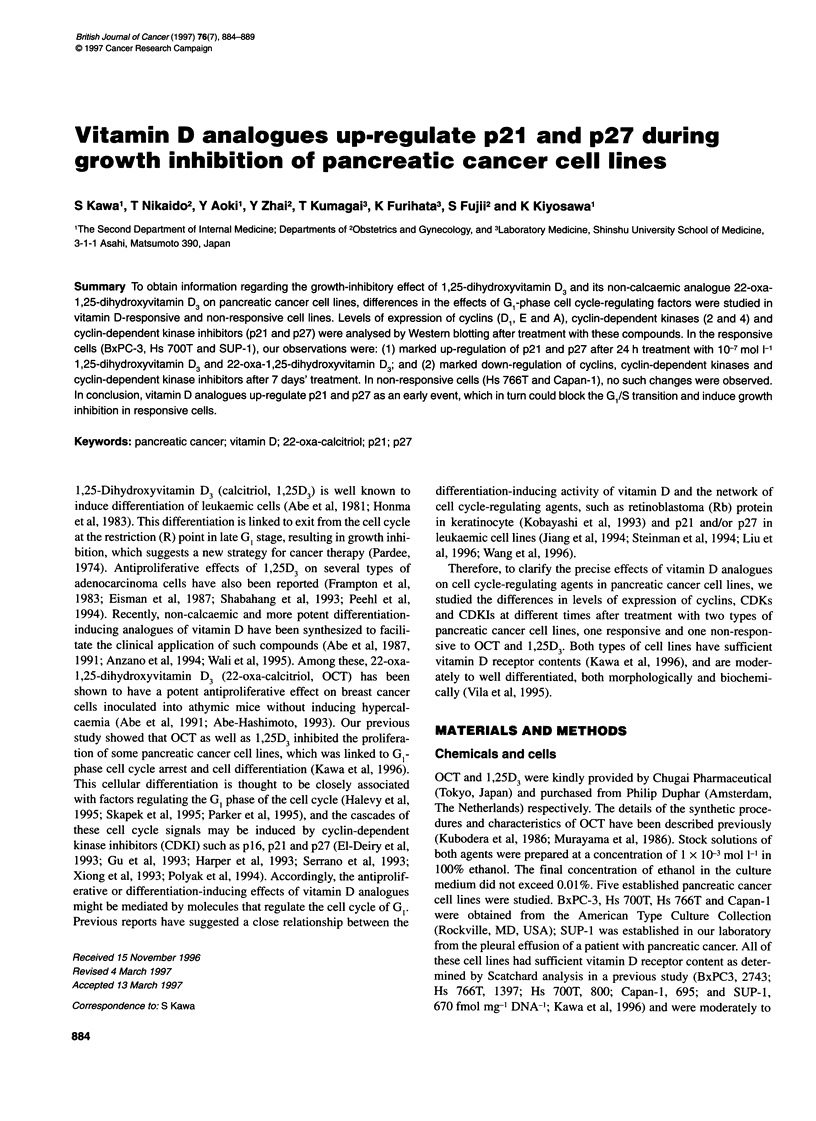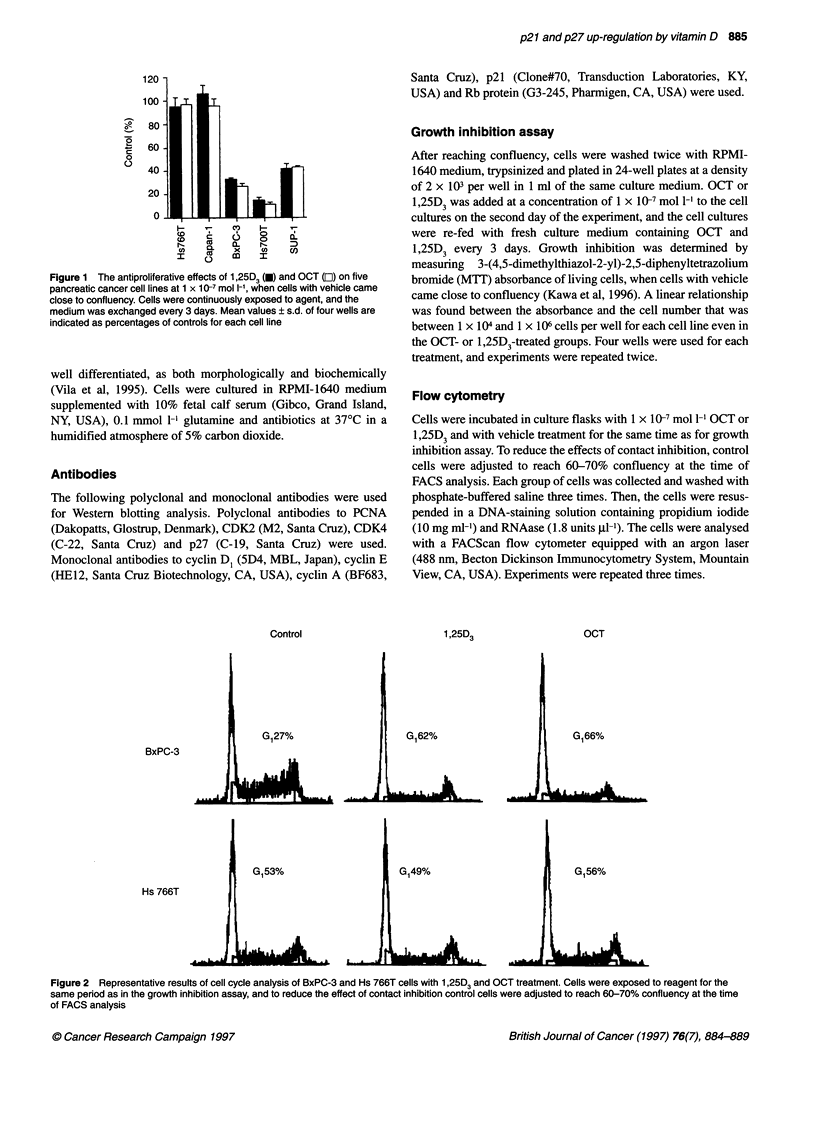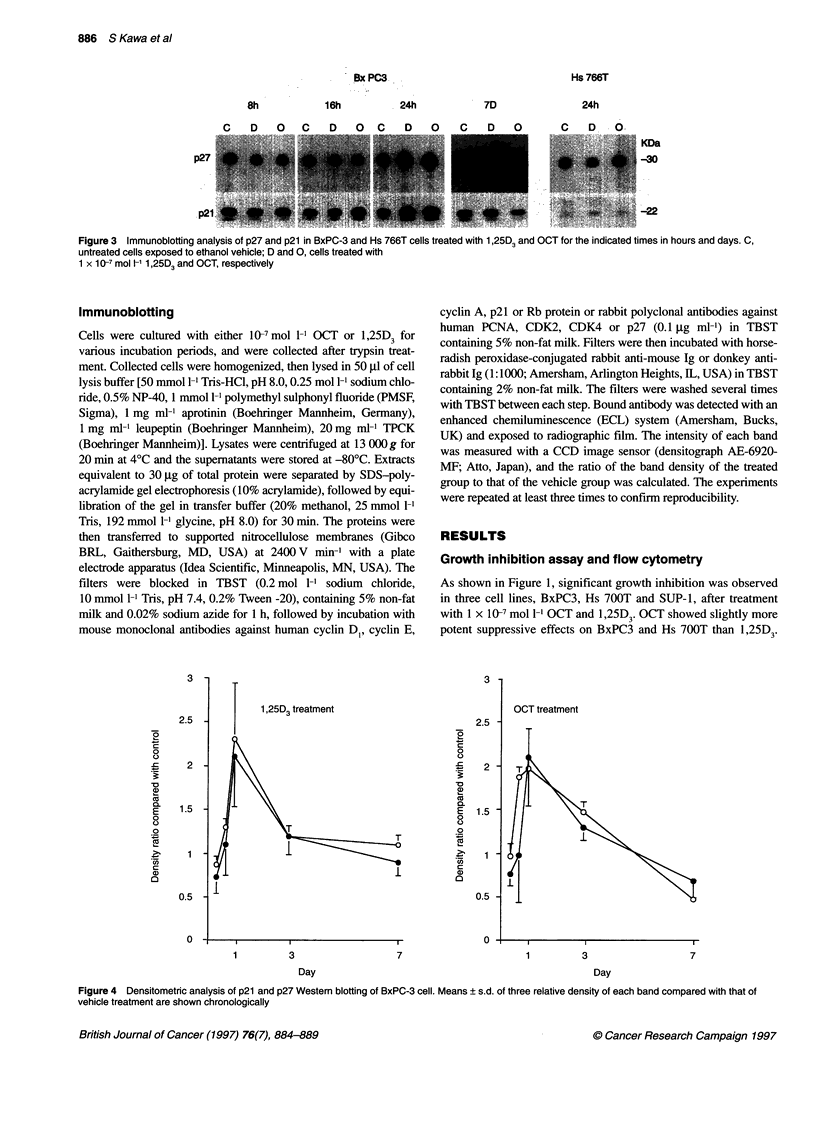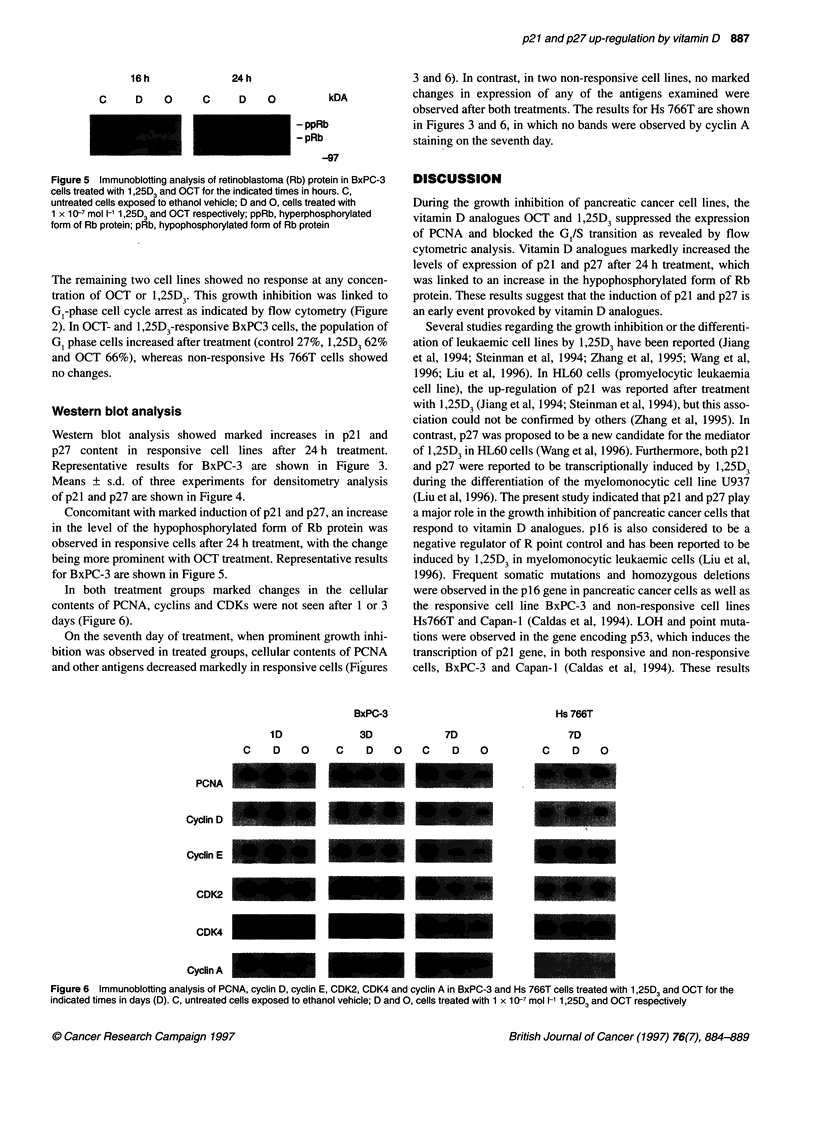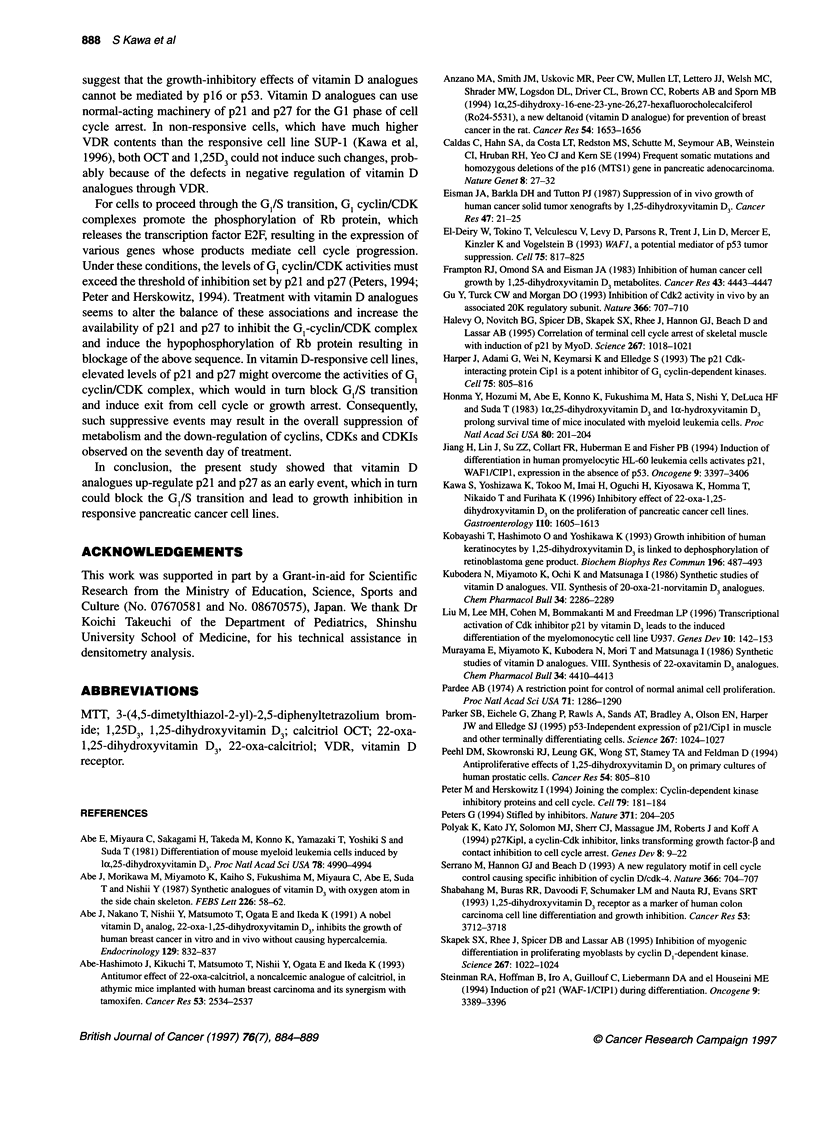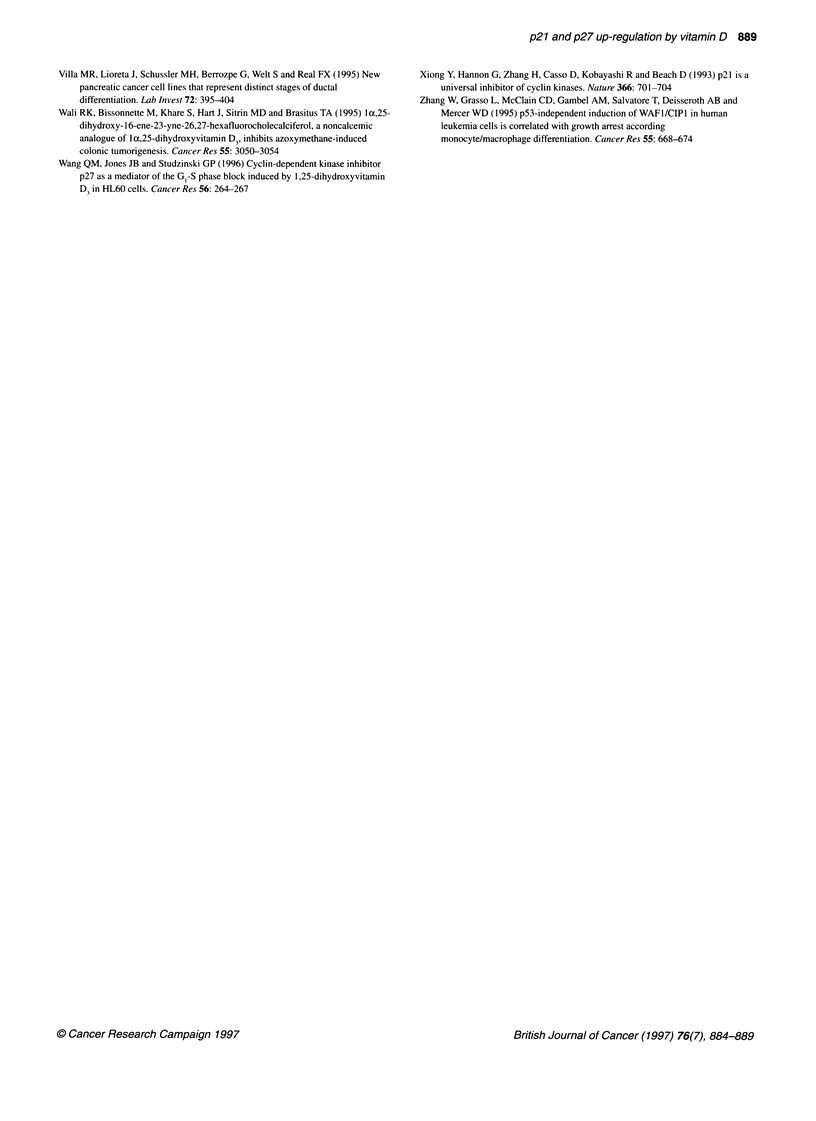# Vitamin D analogues up-regulate p21 and p27 during growth inhibition of pancreatic cancer cell lines.

**DOI:** 10.1038/bjc.1997.479

**Published:** 1997

**Authors:** S. Kawa, T. Nikaido, Y. Aoki, Y. Zhai, T. Kumagai, K. Furihata, S. Fujii, K. Kiyosawa

**Affiliations:** The Second Department of Internal Medicine, Shinshu University School of Medicine, Matsumoto, Japan.

## Abstract

**Images:**


*British Journal of Cancer* (1997) **76**(7), 884–889
© 1997 Cancer Research Campaign

### Vitamin D analogues up-regulate p21 and p27 during growth inhibition of pancreatic cancer cell lines

**S Kawa[1], T Nikaido[2], Y Aoki[1], Y Zhai[2], T Kumagai[3], K Furihata[3], S Fujii[2] and K Kiyosawa[1]**

[1]The Second Department of Internal Medicine; Departments of [2]Obstetrics and Gynecology, and [3]Laboratory Medicine, Shinshu University School of Medicine, 3-1-1 Asahi, Matsumoto 390, Japan

**Summary** To obtain information regarding the growth-inhibitory effect of 1,25-dihydroxyvitamin $D_3$ and its non-calcaemic analogue 22-oxa-1,25-dihydroxyvitamin $D_3$ on pancreatic cancer cell lines, differences in the effects of $G_1$-phase cell cycle-regulating factors were studied in vitamin D-responsive and non-responsive cell lines. Levels of expression of cyclins ($D_1$, E and A), cyclin-dependent kinases (2 and 4) and cyclin-dependent kinase inhibitors (p21 and p27) were analysed by Western blotting after treatment with these compounds. In the responsive cells (BxPC-3, Hs 700T and SUP-1), our observations were: (1) marked up-regulation of p21 and p27 after 24 h treatment with $10^{-7}$ mol $l^{-1}$ 1,25-dihydroxyvitamin $D_3$ and 22-oxa-1,25-dihydroxyvitamin $D_3$; and (2) marked down-regulation of cyclins, cyclin-dependent kinases and cyclin-dependent kinase inhibitors after 7 days' treatment. In non-responsive cells (Hs 766T and Capan-1), no such changes were observed. In conclusion, vitamin D analogues up-regulate p21 and p27 as an early event, which in turn could block the $G_1$/S transition and induce growth inhibition in responsive cells.

**Keywords:** pancreatic cancer; vitamin D; 22-oxa-calcitriol; p21; p27

1,25-Dihydroxyvitamin $D_3$ (calcitriol, 1,25$D_3$) is well known to induce differentiation of leukaemic cells (Abe et al, 1981; Honma et al, 1983). This differentiation is linked to exit from the cell cycle at the restriction (R) point in late $G_1$ stage, resulting in growth inhibition, which suggests a new strategy for cancer therapy (Pardee, 1974). Antiproliferative effects of 1,25$D_3$ on several types of adenocarcinoma cells have also been reported (Frampton et al, 1983; Eisman et al, 1987; Shabahang et al, 1993; Peehl et al, 1994). Recently, non-calcaemic and more potent differentiation-inducing analogues of vitamin D have been synthesized to facilitate the clinical application of such compounds (Abe et al, 1987, 1991; Anzano et al, 1994; Wali et al, 1995). Among these, 22-oxa-1,25-dihydroxyvitamin $D_3$ (22-oxa-calcitriol, OCT) has been shown to have a potent antiproliferative effect on breast cancer cells inoculated into athymic mice without inducing hypercalcaemia (Abe et al, 1991; Abe-Hashimoto, 1993). Our previous study showed that OCT as well as 1,25$D_3$ inhibited the proliferation of some pancreatic cancer cell lines, which was linked to $G_1$-phase cell cycle arrest and cell differentiation (Kawa et al, 1996). This cellular differentiation is thought to be closely associated with factors regulating the $G_1$ phase of the cell cycle (Halevy et al, 1995; Skapek et al, 1995; Parker et al, 1995), and the cascades of these cell cycle signals may be induced by cyclin-dependent kinase inhibitors (CDKI) such as p16, p21 and p27 (El-Deiry et al, 1993; Gu et al, 1993; Harper et al, 1993; Serrano et al, 1993; Xiong et al, 1993; Polyak et al, 1994). Accordingly, the antiproliferative or differentiation-inducing effects of vitamin D analogues might be mediated by molecules that regulate the cell cycle of $G_1$. Previous reports have suggested a close relationship between the differentiation-inducing activity of vitamin D and the network of cell cycle-regulating agents, such as retinoblastoma (Rb) protein in keratinocyte (Kobayashi et al, 1993) and p21 and/or p27 in leukaemic cell lines (Jiang et al, 1994; Steinman et al, 1994; Liu et al, 1996; Wang et al, 1996).

Therefore, to clarify the precise effects of vitamin D analogues on cell cycle-regulating agents in pancreatic cancer cell lines, we studied the differences in levels of expression of cyclins, CDKs and CDKIs at different times after treatment with two types of pancreatic cancer cell lines, one responsive and one non-responsive to OCT and 1,25$D_3$. Both types of cell lines have sufficient vitamin D receptor contents (Kawa et al, 1996), and are moderately to well differentiated, both morphologically and biochemically (Vila et al, 1995).

*Received 15 November 1996*
*Revised 4 March 1997*
*Accepted 13 March 1997*

*Correspondence to:* S Kawa

### MATERIALS AND METHODS

### Chemicals and cells

OCT and 1,25$D_3$ were kindly provided by Chugai Pharmaceutical (Tokyo, Japan) and purchased from Philip Duphar (Amsterdam, The Netherlands) respectively. The details of the synthetic procedures and characteristics of OCT have been described previously (Kubodera et al, 1986; Murayama et al, 1986). Stock solutions of both agents were prepared at a concentration of $1 \times 10^{-3}$ mol $l^{-1}$ in 100% ethanol. The final concentration of ethanol in the culture medium did not exceed 0.01%. Five established pancreatic cancer cell lines were studied. BxPC-3, Hs 700T, Hs 766T and Capan-1 were obtained from the American Type Culture Collection (Rockville, MD, USA); SUP-1 was established in our laboratory from the pleural effusion of a patient with pancreatic cancer. All of these cell lines had sufficient vitamin D receptor content as determined by Scatchard analysis in a previous study (BxPC3, 2743; Hs 766T, 1397; Hs 700T, 800; Capan-1, 695; and SUP-1, 670 fmol $mg^{-1}$ $DNA^{-1}$; Kawa et al, 1996) and were moderately to

**Figure 1** The antiproliferative effects of $1,25D_3$ (■) and OCT (□) on five pancreatic cancer cell lines at $1 \times 10^{-7}$ mol $l^{-1}$, when cells with vehicle came close to confluency. Cells were continuously exposed to agent, and the medium was exchanged every 3 days. Mean values ± s.d. of four wells are indicated as percentages of controls for each cell line well differentiated, as both morphologically and biochemically (Vila et al, 1995). Cells were cultured in RPMI-1640 medium supplemented with 10% fetal calf serum (Gibco, Grand Island, NY, USA), 0.1 mmol $l^{-1}$ glutamine and antibiotics at 37°C in a humidified atmosphere of 5% carbon dioxide.

### Antibodies

The following polyclonal and monoclonal antibodies were used for Western blotting analysis. Polyclonal antibodies to PCNA (Dakopatts, Glostrup, Denmark), CDK2 (M2, Santa Cruz), CDK4 (C-22, Santa Cruz) and p27 (C-19, Santa Cruz) were used. Monoclonal antibodies to cyclin $D_1$ (5D4, MBL, Japan), cyclin E (HE12, Santa Cruz Biotechnology, CA, USA), cyclin A (BF683, Santa Cruz), p21 (Clone#70, Transduction Laboratories, KY, USA) and Rb protein (G3-245, Pharmigen, CA, USA) were used.

### Growth inhibition assay

After reaching confluency, cells were washed twice with RPMI-1640 medium, trypsinized and plated in 24-well plates at a density of $2 \times 10^3$ per well in 1 ml of the same culture medium. OCT or $1,25D_3$ was added at a concentration of $1 \times 10^{-7}$ mol $l^{-1}$ to the cell cultures on the second day of the experiment, and the cell cultures were re-fed with fresh culture medium containing OCT and $1,25D_3$ every 3 days. Growth inhibition was determined by measuring 3-(4,5-dimethylthiazol-2-yl)-2,5-diphenyltetrazolium bromide (MTT) absorbance of living cells, when cells with vehicle came close to confluency (Kawa et al, 1996). A linear relationship was found between the absorbance and the cell number that was between $1 \times 10^4$ and $1 \times 10^6$ cells per well for each cell line even in the OCT- or $1,25D_3$-treated groups. Four wells were used for each treatment, and experiments were repeated twice.

### Flow cytometry

Cells were incubated in culture flasks with $1 \times 10^{-7}$ mol $l^{-1}$ OCT or $1,25D_3$ and with vehicle treatment for the same time as for growth inhibition assay. To reduce the effects of contact inhibition, control cells were adjusted to reach 60–70% confluency at the time of FACS analysis. Each group of cells was collected and washed with phosphate-buffered saline three times. Then, the cells were resuspended in a DNA-staining solution containing propidium iodide (10 mg $ml^{-1}$) and RNAase (1.8 units $\mu l^{-1}$). The cells were analysed with a FACScan flow cytometer equipped with an argon laser (488 nm, Becton Dickinson Immunocytometry System, Mountain View, CA, USA). Experiments were repeated three times.

**Figure 2** Representative results of cell cycle analysis of BxPC-3 and Hs 766T cells with $1,25D_3$ and OCT treatment. Cells were exposed to reagent for the same period as in the growth inhibition assay, and to reduce the effect of contact inhibition control cells were adjusted to reach 60–70% confluency at the time of FACS analysis

© Cancer Research Campaign 1997

**Figure 3** Immunoblotting analysis of p27 and p21 in BxPC-3 and Hs 766T cells treated with $1,25D_3$ and OCT for the indicated times in hours and days. C, untreated cells exposed to ethanol vehicle; D and O, cells treated with $1 \times 10^{-7}$ mol $l^{-1}$ $1,25D_3$ and OCT, respectively

### Immunoblotting

Cells were cultured with either $10^{-7}$ mol $l^{-1}$ OCT or $1,25D_3$ for various incubation periods, and were collected after trypsin treatment. Collected cells were homogenized, then lysed in 50 μl of cell lysis buffer [50 mmol $l^{-1}$ Tris-HCl, pH 8.0, 0.25 mol $l^{-1}$ sodium chloride, 0.5% NP-40, 1 mmol $l^{-1}$ polymethyl sulphonyl fluoride (PMSF, Sigma), 1 mg $ml^{-1}$ aprotinin (Boehringer Mannheim, Germany), 1 mg $ml^{-1}$ leupeptin (Boehringer Mannheim), 20 mg $ml^{-1}$ TPCK (Boehringer Mannheim)]. Lysates were centrifuged at 13 000 *g* for 20 min at 4°C and the supernatants were stored at –80°C. Extracts equivalent to 30 μg of total protein were separated by SDS–polyacrylamide gel electrophoresis (10% acrylamide), followed by equilibration of the gel in transfer buffer (20% methanol, 25 mmol $l^{-1}$ Tris, 192 mmol $l^{-1}$ glycine, pH 8.0) for 30 min. The proteins were then transferred to supported nitrocellulose membranes (Gibco BRL, Gaithersburg, MD, USA) at 2400 V $min^{-1}$ with a plate electrode apparatus (Idea Scientific, Minneapolis, MN, USA). The filters were blocked in TBST (0.2 mol $l^{-1}$ sodium chloride, 10 mmol $l^{-1}$ Tris, pH 7.4, 0.2% Tween -20), containing 5% non-fat milk and 0.02% sodium azide for 1 h, followed by incubation with mouse monoclonal antibodies against human cyclin $D_1$, cyclin E, cyclin A, p21 or Rb protein or rabbit polyclonal antibodies against human PCNA, CDK2, CDK4 or p27 (0.1 μg $ml^{-1}$) in TBST containing 5% non-fat milk. Filters were then incubated with horseradish peroxidase-conjugated rabbit anti-mouse Ig or donkey anti-rabbit Ig (1:1000; Amersham, Arlington Heights, IL, USA) in TBST containing 2% non-fat milk. The filters were washed several times with TBST between each step. Bound antibody was detected with an enhanced chemiluminescence (ECL) system (Amersham, Bucks, UK) and exposed to radiographic film. The intensity of each band was measured with a CCD image sensor (densitograph AE-6920-MF; Atto, Japan), and the ratio of the band density of the treated group to that of the vehicle group was calculated. The experiments were repeated at least three times to confirm reproducibility.

### RESULTS

### Growth inhibition assay and flow cytometry

As shown in Figure 1, significant growth inhibition was observed in three cell lines, BxPC3, Hs 700T and SUP-1, after treatment with $1 \times 10^{-7}$ mol $l^{-1}$ OCT and $1,25D_3$. OCT showed slightly more potent suppressive effects on BxPC3 and Hs 700T than $1,25D_3$.

**Figure 4** Densitometric analysis of p21 and p27 Western blotting of BxPC-3 cell. Means ± s.d. of three relative density of each band compared with that of vehicle treatment are shown chronologically

 *© Cancer Research Campaign 1997*

**Figure 5** Immunoblotting analysis of retinoblastoma (Rb) protein in BxPC-3 cells treated with 1,25$D_3$ and OCT for the indicated times in hours. C, untreated cells exposed to ethanol vehicle; D and O, cells treated with $1 \times 10^{-7}$ mol $l^{-1}$ 1,25$D_3$ and OCT respectively; ppRb, hyperphosphorylated form of Rb protein; pRb, hypophosphorylated form of Rb protein

The remaining two cell lines showed no response at any concentration of OCT or 1,25$D_3$. This growth inhibition was linked to $G_1$-phase cell cycle arrest as indicated by flow cytometry (Figure 2). In OCT- and 1,25$D_3$-responsive BxPC3 cells, the population of $G_1$ phase cells increased after treatment (control 27%, 1,25$D_3$ 62% and OCT 66%), whereas non-responsive Hs 766T cells showed no changes.

### Western blot analysis

Western blot analysis showed marked increases in p21 and p27 content in responsive cell lines after 24 h treatment. Representative results for BxPC-3 are shown in Figure 3. Means ± s.d. of three experiments for densitometry analysis of p21 and p27 are shown in Figure 4.

Concomitant with marked induction of p21 and p27, an increase in the level of the hypophosphorylated form of Rb protein was observed in responsive cells after 24 h treatment, with the change being more prominent with OCT treatment. Representative results for BxPC-3 are shown in Figure 5.

In both treatment groups marked changes in the cellular contents of PCNA, cyclins and CDKs were not seen after 1 or 3 days (Figure 6).

On the seventh day of treatment, when prominent growth inhibition was observed in treated groups, cellular contents of PCNA and other antigens decreased markedly in responsive cells (Figures 3 and 6). In contrast, in two non-responsive cell lines, no marked changes in expression of any of the antigens examined were observed after both treatments. The results for Hs 766T are shown in Figures 3 and 6, in which no bands were observed by cyclin A staining on the seventh day.

### DISCUSSION

During the growth inhibition of pancreatic cancer cell lines, the vitamin D analogues OCT and 1,25$D_3$ suppressed the expression of PCNA and blocked the $G_1$/S transition as revealed by flow cytometric analysis. Vitamin D analogues markedly increased the levels of expression of p21 and p27 after 24 h treatment, which was linked to an increase in the hypophosphorylated form of Rb protein. These results suggest that the induction of p21 and p27 is an early event provoked by vitamin D analogues.

Several studies regarding the growth inhibition or the differentiation of leukaemic cell lines by 1,25$D_3$ have been reported (Jiang et al, 1994; Steinman et al, 1994; Zhang et al, 1995; Wang et al, 1996; Liu et al, 1996). In HL60 cells (promyelocytic leukaemia cell line), the up-regulation of p21 was reported after treatment with 1,25$D_3$ (Jiang et al, 1994; Steinman et al, 1994), but this association could not be confirmed by others (Zhang et al, 1995). In contrast, p27 was proposed to be a new candidate for the mediator of 1,25$D_3$ in HL60 cells (Wang et al, 1996). Furthermore, both p21 and p27 were reported to be transcriptionally induced by 1,25$D_3$ during the differentiation of the myelomonocytic cell line U937 (Liu et al, 1996). The present study indicated that p21 and p27 play a major role in the growth inhibition of pancreatic cancer cells that respond to vitamin D analogues. p16 is also considered to be a negative regulator of R point control and has been reported to be induced by 1,25$D_3$ in myelomonocytic leukaemic cells (Liu et al, 1996). Frequent somatic mutations and homozygous deletions were observed in the p16 gene in pancreatic cancer cells as well as the responsive cell line BxPC-3 and non-responsive cell lines Hs766T and Capan-1 (Caldas et al, 1994). LOH and point mutations were observed in the gene encoding p53, which induces the transcription of p21 gene, in both responsive and non-responsive cells, BxPC-3 and Capan-1 (Caldas et al, 1994). These results

**Figure 6** Immunoblotting analysis of PCNA, cyclin D, cyclin E, CDK2, CDK4 and cyclin A in BxPC-3 and Hs 766T cells treated with 1,25$D_3$ and OCT for the indicated times in days (D). C, untreated cells exposed to ethanol vehicle; D and O, cells treated with $1 \times 10^{-7}$ mol $l^{-1}$ 1,25$D_3$ and OCT respectively

© Cancer Research Campaign 1997

suggest that the growth-inhibitory effects of vitamin D analogues cannot be mediated by p16 or p53. Vitamin D analogues can use normal-acting machinery of p21 and p27 for the G1 phase of cell cycle arrest. In non-responsive cells, which have much higher VDR contents than the responsive cell line SUP-1 (Kawa et al, 1996), both OCT and 1,25$D_3$ could not induce such changes, probably because of the defects in negative regulation of vitamin D analogues through VDR.

For cells to proceed through the $G_1$/S transition, $G_1$ cyclin/CDK complexes promote the phosphorylation of Rb protein, which releases the transcription factor E2F, resulting in the expression of various genes whose products mediate cell cycle progression. Under these conditions, the levels of $G_1$ cyclin/CDK activities must exceed the threshold of inhibition set by p21 and p27 (Peters, 1994; Peter and Herskowitz, 1994). Treatment with vitamin D analogues seems to alter the balance of these associations and increase the availability of p21 and p27 to inhibit the $G_1$-cyclin/CDK complex and induce the hypophosphorylation of Rb protein resulting in blockage of the above sequence. In vitamin D-responsive cell lines, elevated levels of p21 and p27 might overcome the activities of $G_1$ cyclin/CDK complex, which would in turn block $G_1$/S transition and induce exit from cell cycle or growth arrest. Consequently, such suppressive events may result in the overall suppression of metabolism and the down-regulation of cyclins, CDKs and CDKIs observed on the seventh day of treatment.

In conclusion, the present study showed that vitamin D analogues up-regulate p21 and p27 as an early event, which in turn could block the $G_1$/S transition and lead to growth inhibition in responsive pancreatic cancer cell lines.

### ACKNOWLEDGEMENTS

This work was supported in part by a Grant-in-aid for Scientific Research from the Ministry of Education, Science, Sports and Culture (No. 07670581 and No. 08670575), Japan. We thank Dr Koichi Takeuchi of the Department of Pediatrics, Shinshu University School of Medicine, for his technical assistance in densitometry analysis.

### ABBREVIATIONS

MTT, 3-(4,5-dimetylthiazol-2-yl)-2,5-diphenyltetrazolium bromide; 1,25$D_3$, 1,25-dihydroxyvitamin $D_3$; calcitriol OCT; 22-oxa-1,25-dihydroxyvitamin $D_3$, 22-oxa-calcitriol; VDR, vitamin D receptor.

### REFERENCES

Abe E, Miyaura C, Sakagami H, Takeda M, Konno K, Yamazaki T, Yoshiki S and Suda T (1981) Differentiation of mouse myeloid leukemia cells induced by 1α,25-dihydroxyvitamin $D_3$. *Proc Natl Acad Sci USA* **78**: 4990–4994

Abe J, Morikawa M, Miyamoto K, Kaiho S, Fukushima M, Miyaura C, Abe E, Suda T and Nishii Y (1987) Synthetic analogues of vitamin $D_3$ with oxygen atom in the side chain skeleton. *FEBS Lett* **226**: 58–62.

Abe J, Nakano T, Nishii Y, Matsumoto T, Ogata E and Ikeda K (1991) A nobel vitamin $D_3$ analog, 22-oxa-1,25-dihydroxyvitamin $D_3$, inhibits the growth of human breast cancer in vitro and in vivo without causing hypercalcemia. *Endocrinology* **129**: 832–837

Abe-Hashimoto J, Kikuchi T, Matsumoto T, Nishii Y, Ogata E and Ikeda K (1993) Antitumor effect of 22-oxa-calcitriol, a noncalcemic analogue of calcitriol, in athymic mice implanted with human breast carcinoma and its synergism with tamoxifen. *Cancer Res* **53**: 2534–2537

Anzano MA, Smith JM, Uskovic MR, Peer CW, Mullen LT, Lettero JJ, Welsh MC, Shrader MW, Logsdon DL, Driver CL, Brown CC, Roberts AB and Sporn MB (1994) 1α,25-dihydroxy-16-ene-23-yne-26,27-hexafluorocholecalciferol (Ro24-5531), a new deltanoid (vitamin D analogue) for prevention of breast cancer in the rat. *Cancer Res* **54**: 1653–1656

Caldas C, Hahn SA, da Costa LT, Redston MS, Schutte M, Seymour AB, Weinstein CI, Hruban RH, Yeo CJ and Kern SE (1994) Frequent somatic mutations and homozygous deletions of the p16 (MTS1) gene in pancreatic adenocarcinoma. *Nature Genet* **8**: 27–32

Eisman JA, Barkla DH and Tutton PJ (1987) Suppression of in vivo growth of human cancer solid tumor xenografts by 1,25-dihydroxyvitamin $D_3$. *Cancer Res* **47**: 21–25

El-Deiry W, Tokino T, Velculescu V, Levy D, Parsons R, Trent J, Lin D, Mercer E, Kinzler K and Vogelstein B (1993) *WAF1*, a potential mediator of p53 tumor suppression. *Cell* **75**: 817–825

Frampton RJ, Omond SA and Eisman JA (1983) Inhibition of human cancer cell growth by 1,25-dihydroxyvitamin $D_3$ metabolites. *Cancer Res* **43**: 4443–4447

Gu Y, Turck CW and Morgan DO (1993) Inhibition of Cdk2 activity in vivo by an associated 20K regulatory subunit. *Nature* **366**: 707–710

Halevy O, Novitch BG, Spicer DB, Skapek SX, Rhee J, Hannon GJ, Beach D and Lassar AB (1995) Correlation of terminal cell cycle arrest of skeletal muscle with induction of p21 by MyoD. *Science* **267**: 1018–1021

Harper J, Adami G, Wei N, Keymarsi K and Elledge S (1993) The p21 Cdk-interacting protein Cip1 is a potent inhibitor of $G_1$ cyclin-dependent kinases. *Cell* **75**: 805–816

Honma Y, Hozumi M, Abe E, Konno K, Fukushima M, Hata S, Nishi Y, DeLuca HF and Suda T (1983) 1α,25-dihydroxyvitamin $D_3$ and 1α-hydroxyvitamin $D_3$ prolong survival time of mice inoculated with myeloid leukemia cells. *Proc Natl Acad Sci USA* **80**: 201–204

Jiang H, Lin J, Su ZZ, Collart FR, Huberman E and Fisher PB (1994) Induction of differentiation in human promyelocytic HL-60 leukemia cells activates p21, WAF1/CIP1, expression in the absence of p53. *Oncogene* **9**: 3397–3406

Kawa S, Yoshizawa K, Tokoo M, Imai H, Oguchi H, Kiyosawa K, Homma T, Nikaido T and Furihata K (1996) Inhibitory effect of 22-oxa-1,25-dihydroxyvitamin $D_3$ on the proliferation of pancreatic cancer cell lines. *Gastroenterology* **110**: 1605–1613

Kobayashi T, Hashimoto O and Yoshikawa K (1993) Growth inhibition of human keratinocytes by 1,25-dihydroxyvitamin $D_3$ is linked to dephosphorylation of retinoblastoma gene product. *Biochem Biophys Res Commun* **196**: 487–493

Kubodera N, Miyamoto K, Ochi K and Matsunaga I (1986) Synthetic studies of vitamin D analogues. VII. Synthesis of 20-oxa-21-norvitamin $D_3$ analogues. *Chem Pharmacol Bull* **34**: 2286–2289

Liu M, Lee MH, Cohen M, Bommakanti M and Freedman LP (1996) Transcriptional activation of Cdk inhibitor p21 by vitamin $D_3$ leads to the induced differentiation of the myelomonocytic cell line U937. *Genes Dev* **10**: 142–153

Murayama E, Miyamoto K, Kubodera N, Mori T and Matsunaga I (1986) Synthetic studies of vitamin D analogues. VIII. Synthesis of 22-oxavitamin $D_3$ analogues. *Chem Pharmacol Bull* **34**: 4410–4413

Pardee AB (1974) A restriction point for control of normal animal cell proliferation. *Proc Natl Acad Sci USA* **71**: 1286–1290

Parker SB, Eichele G, Zhang P, Rawls A, Sands AT, Bradley A, Olson EN, Harper JW and Elledge SJ (1995) p53-Independent expression of p21/Cip1 in muscle and other terminally differentiating cells. *Science* **267**: 1024–1027

Peehl DM, Skowronski RJ, Leung GK, Wong ST, Stamey TA and Feldman D (1994) Antiproliferative effects of 1,25-dihydroxyvitamin $D_3$ on primary cultures of human prostatic cells. *Cancer Res* **54**: 805–810

Peter M and Herskowitz I (1994) Joining the complex: Cyclin-dependent kinase inhibitory proteins and cell cycle. *Cell* **79**: 181–184

Peters G (1994) Stifled by inhibitors. *Nature* **371**: 204–205

Polyak K, Kato JY, Solomon MJ, Sherr CJ, Massague JM, Roberts J and Koff A (1994) p27Kipl, a cyclin-Cdk inhibitor, links transforming growth factor-β and contact inhibition to cell cycle arrest. *Genes Dev* **8**: 9–22

Serrano M, Hannon GJ and Beach D (1993) A new regulatory motif in cell cycle control causing specific inhibition of cyclin D/cdk-4. *Nature* **366**: 704–707

Shabahang M, Buras RR, Davoodi F, Schumaker LM and Nauta RJ, Evans SRT (1993) 1,25-dihydroxyvitamin $D_3$ receptor as a marker of human colon carcinoma cell line differentiation and growth inhibition. *Cancer Res* **53**: 3712–3718

Skapek SX, Rhee J, Spicer DB and Lassar AB (1995) Inhibition of myogenic differentiation in proliferating myoblasts by cyclin $D_1$-dependent kinase. *Science* **267**: 1022–1024

Steinman RA, Hoffman B, Iro A, Guillouf C, Liebermann DA and el Houseini ME (1994) Induction of p21 (WAF-1/CIP1) during differentiation. *Oncogene* **9**: 3389–3396

© Cancer Research Campaign 1997

Villa MR, Lioreta J, Schussler MH, Berrozpe G, Welt S and Real FX (1995) New pancreatic cancer cell lines that represent distinct stages of ductal differentiation. *Lab Invest* **72**: 395–404

Wali RK, Bissonnette M, Khare S, Hart J, Sitrin MD and Brasitus TA (1995) 1α,25-dihydroxy-16-ene-23-yne-26,27-hexafluorocholecalciferol, a noncalcemic analogue of 1α,25-dihydroxyvitamin $D_3$, inhibits azoxymethane-induced colonic tumorigenesis. *Cancer Res* **55**: 3050–3054

Wang QM, Jones JB and Studzinski GP (1996) Cyclin-dependent kinase inhibitor p27 as a mediator of the $G_1$-S phase block induced by 1,25-dihydroxyvitamin $D_3$ in HL60 cells. *Cancer Res* **56**: 264–267

Xiong Y, Hannon G, Zhang H, Casso D, Kobayashi R and Beach D (1993) p21 is a universal inhibitor of cyclin kinases. *Nature* **366**: 701–704

Zhang W, Grasso L, McClain CD, Gambel AM, Salvatore T, Deisseroth AB and Mercer WD (1995) p53-independent induction of WAF1/CIP1 in human leukemia cells is correlated with growth arrest according monocyte/macrophage differentiation. *Cancer Res* **55**: 668–674

© Cancer Research Campaign 1997